# A pedometer-based walking intervention with and without email counseling in general practice: a pilot randomized controlled trial

**DOI:** 10.1186/s12889-018-5520-8

**Published:** 2018-05-16

**Authors:** Tomas Vetrovsky, Jozef Cupka, Martin Dudek, Blanka Kuthanova, Klaudia Vetrovska, Vaclav Capek, Vaclav Bunc

**Affiliations:** 1 Faculty of Physical Education and Sport, Charles University, Jose Martiho 31, 162 52 Prague 6, Czech Republic; 2 Mediciman s.r.o, Maxovska 1019/6, 155 00 Prague 5, Czech Republic; 3 Laureus s.r.o, Palackeho 541, 252 29 Dobrichovice, Czech Republic; 4 Praktici Praha 6, s.r.o, Vitezne namesti 817/9, 160 00 Prague 6, Czech Republic; 5 Humilitas s.r.o, Na Veselou 698/2, 266 01 Beroun, Czech Republic; 6 Second Faculty of Medicine, Charles University, V Uvalu 84, 150 06 Prague 5, Czech Republic

**Keywords:** Primary care, Pedometer, Email counseling, Walking, Physical activity, General practitioner, Recruitment, Adherence, Qualitative research

## Abstract

**Background:**

General practitioners play a fundamental role in combatting the current epidemic of physical inactivity, and pedometer-based walking interventions are able to increase physical activity levels of their patients. Supplementing these interventions with email counseling driven by feedback from the pedometer has the potential to further improve their effectiveness but it has to be yet confirmed in clinical trials. Therefore, the aim of our pilot randomized controlled trial is to evaluate the feasibility and potential efficacy of future trials designed to assess the additional benefit of email counseling added to a pedometer-based intervention in a primary care setting.

**Methods:**

Physically inactive patients were opportunistically recruited from four general practices and randomized to a 12-week pedometer-based intervention with or without email counseling. To explore the feasibility of future trials, we assessed the speed and efficiency of recruitment, adherence to wearing the pedometer, and engagement with email counseling. To evaluate the potential efficacy, daily step-count was the primary outcome and blood pressure, waist and hip circumference, and body mass were the secondary outcomes. Additionally, we conducted a qualitative analysis of structured interviews with the participating general practitioners.

**Results:**

The opportunistic recruitment has been shown to be feasible and acceptable, but relatively slow and inefficient; moreover, general practitioners selectively recruited overweight and obese patients. Patients manifested high adherence, wearing the pedometer on 83% (± 20) of days. All patients from the counseling group actively participated in email communication and responded to 46% (± 22) of the emails they received. Both groups significantly increased their daily step-count (pedometer-plus-email, + 2119, *p* = 0.002; pedometer-alone, + 1336, *p* = 0.03), but the difference between groups was not significant (*p* = 0.18). When analyzing both groups combined, there was a significant decrease in body mass (− 0.68 kg, *p* = 0.04), waist circumference (− 1.73 cm, *p* = 0.03), and systolic blood pressure (− 3.48 mmHg, *p* = 0.045).

**Conclusions:**

This study demonstrates that adding email counseling to a pedometer-based intervention in a primary care setting is feasible and might have the potential to increase the efficacy of such an intervention in increasing physical activity levels.

**Trial registration:**

The trial was retrospectively registered at ClinicalTrials.gov (ID: NCT03135561, date: April 26, 2017).

## Background

Insufficient physical activity (PA) is one of the leading modifiable risk factors responsible for numerous chronic diseases and for premature death [1–4]. As 70–80% of adults in developed countries visit their general practitioner (GP) at least once a year [5], GPs are well situated to deliver PA interventions to physically inactive adults [6, 7]. Moreover, most GPs believe that PA counseling is important and that they play a role in promoting PA among their patients [8]. In addition, GPs are generally viewed as being credible sources of health information, particularly among older adults and those with multiple chronic diseases [9]. Thus, it is not surprising that the National Institute for Health and Care Excellence in the UK recommends that GPs should identify inactive adults and advise them to increase their PA levels [10].

Walking can be considered as the most natural form of PA as it is easily performed by everyone except for the seriously disabled or very frail. As such, walking can be easily incorporated into many activities of daily living and has been the main option for increasing PA in sedentary populations [11]. Interventions aimed at promoting walking could substantially contribute towards increasing PA levels of the most sedentary individuals and serve as an important cornerstone in many PA promotional campaigns [12]. Within these interventions, pedometers are commonly used as effective motivational instruments to increase walking in healthy adults and across a range of clinical conditions [13–18].

In spite of the well documented ability of pedometer-based walking interventions to increase PA levels, their effectiveness in primary care settings is far from optimal [19–23] due to both patient- and provider-related factors. Though patients perceive self-monitoring with pedometers as motivating, their efforts to increase PA levels are often hindered by substantial barriers such as inflexible work routines, long working hours, domestic duties, suboptimal weather conditions, a lack of motivation, and other commitments [24–26]. Despite these barriers, GPs consider pedometers to be helpful for increasing PA levels of their patients, but they often lack the time and appropriate training necessary to deliver pedometer-based PA interventions [8, 27].

Hence, there remains a need for further improvement of pedometer-based interventions in primary care settings, possibly by adding a counseling component that could be delivered face-to-face, over the telephone, or via the internet [28–30]. Counseling provided in regular intervals throughout the intervention period could positively influence patients’ adherence, and employing effective behavioral techniques during counseling could help a patient overcome certain psychological or lifestyle barriers, ultimately increasing PA. Moreover, such counseling can be performed by a trained counselor outside normal office hours, thus reducing the burden on the GP [30].

Several studies investigated the effects of a pedometer-plus-counseling intervention, however they compared it to either a usual care group [19, 20] or a counseling-alone group [22, 31], not allowing the effects of pedometer-plus-counseling to be compared to only a pedometer. Additionally, those few studies that have directly compared pedometer-based interventions with and without counseling in primary care settings [21, 32–34] gave inconclusive results. Currently, a handful of ongoing studies have combined a pedometer with some form of face-to-face or phone counseling in primary [35, 36] and secondary [37, 38] care settings, but their results are not yet publicly available.

Considering the various types of counseling that can be used to communicate with patients, email counseling may be more effective than traditional face-to-face and telephone counseling, as it gives both patients and counselors greater flexibility regarding when and where the interactions occur. Indeed, email counseling has been demonstrated to be effective in various health behavior interventions such as reducing fatigue in multiple sclerosis patients [39], achieving weight loss in overweight adults [40–42], or improving diet in college students [43].

Also, email communication has long been used in internet-based PA interventions [44–47], but it is usually employed only as a channel for one-way message delivery from the researcher to the participant [48, 49] or as a reminder to encourage participants to visit a web-based intervention program [50]. Few studies have used email as a tool for delivering two-way interactive PA counseling [51, 52], and studies combining email counseling with personalized feedback based on objectively measured PA using pedometers are practically non-existent.

Therefore, the aim of this pilot randomized controlled trial was to assess feasibility and to support the development of future trials in a primary care setting, designed to assess the additional benefit of email counseling added to a pedometer-based intervention. The specific objectives were to: (a) explore the feasibility of the recruitment procedure, (b) evaluate patients’ adherence to the interventions, (c) examine patients’ engagement with the email counseling, (d) assess the potential efficacy of the interventions on daily step counts and other health-related outcomes. In addition, we conducted a qualitative analysis of structured interviews with the participating GPs to gain more insight into the feasibility of the trial and how to improve it.

## Methods

### Design and settings

**Fig. 1:**
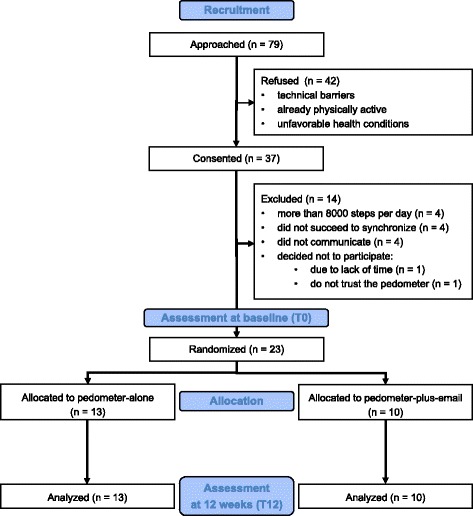
CONSORT 2010 flow diagram

A two-arm parallel pilot randomized controlled trial comparing a pedometer-based intervention with and without email counseling was conducted in four general practices across the Czech Republic. Recruitment started in November 2015 and was completed in June 2016. Outcomes were assessed at baseline and 12 weeks post-randomization. A CONSORT flow diagram of the progress through the phases of the study is illustrated in Fig. 1 [53].

The study was reviewed and approved by the ethics committee of the Faculty of Physical Education and Sports, Charles University (081/2015), and it was conducted according to the principles of the Declaration of Helsinki. Eligible patients were informed about all relevant aspects of the study before enrolling, notified about the right to refuse to participate or to withdraw consent at any time without reprisal, and then provided written informed consent. The trial was retrospectively registered at ClinicalTrials.gov (ID: NCT03135561, date: April 26, 2017).

### Participants and enrollment

Patients were opportunistically recruited from four general practices that were selected to represent a large city, a middle-sized town, and a small town in the Czech Republic. The GPs, who are co-investigators in this trial, approached patients during routine preventive health checkups, screened them for eligibility, introduced the study to the eligible subjects, and obtained written informed consent from those who were interested. The GPs also maintained a log where all excluded patients were recorded, noting the reasons why there were excluded.

Patients were eligible if they met all of the following inclusion criteria: (1) registered at a selected general practice, (2) provided written informed consent before any assessment related to the study, (3) were over 18 years of age, (4) identified themselves as regular email users, and were willing to use email as part of the study, (5) had a home computer with access to the internet, (6) were physically inactive, as determined by a negative response to the following question: “As a rule, do you do at least half an hour of moderate or vigorous exercise (such as walking or a sport) on five or more days of the week?”. This screening question has a high positive predictive value (86.7%) for identifying individuals who do not achieve the recommended 150 min of moderate level PA per week [54].

Patients were excluded if they: (1) had co-morbid conditions that would affect adherence to trial procedures (e.g. inflammatory arthritis, active malignancy, renal disease requiring dialysis, uncontrolled diabetes, major depression or other significant psychiatric disorders, dementia or cognitive impairment, significant hearing or visual impairment, or a terminal illness), (2) had a medical, personal, or family condition which the GP considered to affect mean daily step count at baseline (e.g., acute illness, holiday or business trip), (3) were unable to walk for any reason, (4) were pregnant women, (5) were currently engaging in regular sports or exercise (at least twice a week), (6) were already tracking their steps with their own device, or (7) were achieving 8000 steps or more at the baseline assessment.

After signing the informed consent during the same initial GP visit, anthropometric measures and resting blood pressure were assessed. Finally, participants received a pedometer blinded with adhesive tape, were instructed to wear it on their neck for 7 full days during waking hours except when swimming or bathing, and were told to not change their usual PA levels. After 7 days, participants were requested to remove the adhesive tape and upload the data to a website for viewing online.

Following the upload of pedometer data, mean daily step count from the 7 days was calculated for each participant, and those with a mean daily step count lower than 8000 were randomized to either a pedometer-alone (PED) or pedometer-plus-email (PEMAIL) group at a 1:1 ratio. Patient allocation was performed using a free online tool at http://www.sealedenvelope.com, using a permuted block randomization scheme stratified by practice. Participants who failed to upload pedometer data and those whose mean daily step count was 8000 or more were excluded from the study.

It was not possible to blind the participants or researchers since both were naturally aware of the group allocation due to their active roles in the intervention. However, post-intervention assessments were undertaken by a nurse who was blinded to the group allocation.

### Interventions

Once randomized, all participants were informed of their allocated group by an email from the main researcher. In this email, all participants were instructed to wear the pedometer around the neck daily for the next 4 months, check the step count every evening, and gradually increase the daily number of steps up to 10,000. They were also required to upload data to a website at least once a week and were encouraged to contact technical support if they experienced problems with uploading the data.

### PED group

The eVito 3D Step Counter SL three-dimensional pedometer (HMM Diagnostics GmbH, Dossenheim, Germany) was chosen for the intervention as it features three-dimensional accelerometers to record the number of steps made per minute, memory to store data for more than 30 days, and ANT+ wireless technology to upload data to a website where data could be viewed online by the participants or a member of the research team.

This pedometer can be worn in the pockets, on the belt, or around the neck. For the purpose of this study we instructed participants to wear it around the neck, as this location has been shown to be highly accurate and preferred by participants [55]. We assessed the validity and reliability of the eVito 3D Step Counter SL pedometer across several velocities (3.0, 3.6, 4.2 kph) on a treadmill and during six-minute walk test in a laboratory corridor by using visually counted steps as a criterion (mean absolute percentage error between 1.3% and 5.6; Pearson correlation coefficient between 0.62 and 0.99).

Participants in the PED group were only contacted if they failed to upload the pedometer data for more than 2 weeks. In that case, they were sent a brief email reminder to do so. Apart from checking the pedometer every evening and trying to increase the daily step count up to 10,000 steps, they received no further instructions or specific goals.

### PEMAIL group

Participants in the PEMAIL group received the same pedometer and instructions as those in the PED group. In addition, the main researcher, trained in behavioral techniques, communicated with them regularly during the 12-week intervention period via email using effective behavioral principles [13, 56, 57] that were focused on helping the participants achieve their daily step goals. Self-monitoring, action planning, goal setting, and personalized feedback were the key techniques used in the intervention.

During the first 4 weeks of the intervention, the participants were sent emails on a weekly basis. For the remaining 8 weeks, emails were sent on a bi-weekly basis. The last email was sent at least 10 days before the assessment period to avoid immediate reactivity. Altogether, eight counseling emails were sent during the intervention period.

In the first counseling email, participants were set an individual progressive goal expressed as a weekly increase in the daily number of steps, determined as 15% of the subject’s baseline value rounded to nearest hundred. For example, a participant with a baseline value of 4000 steps per day was recommended to increase the daily step number by 600 each week, aiming for at least 10,000 steps a day. The participants were asked to suggest their own strategies to achieve this goal by identifying opportunities in their daily routine when they could include at least a 10-min walk (e.g., park farther away, walk to/from lunch, walk before/after work).

The subsequent emails were drafted individually, tailored to the specific needs of the participant and the circumstances of their case, and meant to elicit their response. Whenever a participant responded to an email, the subsequent email from the researcher was drafted as a response to the participant’s email, thus giving the feeling of a natural email conversation.

Although individual, the emails always incorporated some common features: (a) encouragement of the participants based on their objectively measured achievement in the previous week, (b) reminder of the benefits of PA for the physical and mental health relevant to the individual participant, (c) discussion of individual behavioral strategies, what works for them, and what does not, and (d) setting of the goal for the upcoming week.

### Outcome measures

#### Feasibility of the recruitment procedure

To evaluate the feasibility of the recruitment procedure, we assessed the speed of recruitment (expressed as the number of patients per week of the active recruitment period per general practice), and efficiency of the recruitment (expressed as the ratio of randomized to recruited patients).

#### Patients’ adherence and engagement

The percentage of valid days was calculated as a measure of patients’ adherence to wearing the pedometer. For the purposes of this study, a valid day was defined as one with at least 8 h with a step count above zero. Periods with known technical issues related to the pedometer were excluded from this analysis. The percentage of patients who completed the study was also evaluated and reasons of discontinuation were identified. Additionally, in the PEMAIL group, the percentage of patient email responses to the counselor’s emails was calculated to express patient engagement.

#### Potential efficacy of the interventions

Though this was a pilot study that was not adequately powered to assess differences between groups, we still aimed to evaluate the potential efficacy of the interventions for the purpose of the power analysis of a future trial. The primary efficacy outcome was a change in mean daily step count from baseline (T0) to 12 weeks post-randomization (T12). The secondary outcomes were the changes from T0 to T12 in systolic and diastolic blood pressure, waist and hip circumference, and body mass. In addition, patient-reported outcomes (health-related quality of life, and depression and anxiety) were collected before and after the intervention for the purpose of a quasi-experimental pre/post study whose results were published separately [58].

The same eVito 3D Step Counter SL pedometer that was used for the intervention in both groups was employed to objectively measure average daily step count. Mean daily step count from the first 7 days of wearing the blinded pedometer was used as a baseline value. The T12 mean daily step count was calculated from the 7-day period starting 84 days after randomization. As participants in both groups were instructed to continually wear pedometers and to regularly upload step data to a website without knowing at which time point their step performance is to be evaluated, we could use their uploaded data as the outcome measure without the risk of a Hawthorne effect, even though the pedometer was not blinded by the adhesive tape at that point.

Body mass, waist and hip circumference, and blood pressure were measured by a practice nurse blinded to the participants’ group allocation. Body mass was measured to the nearest kilogram using a standard calibrated scale available in the GP’s office. Waist and hip circumferences were recorded with a measurement tape to the nearest centimeter, according to established protocols [59]. Blood pressure was assessed using an automated monitor available in the GP’s office.

### Data analysis

Primary and secondary efficacy outcomes were compared between the two groups using a two-sided two-sample *t* test or its non-parametric alternative, if necessary. Changes from baseline to post-intervention were evaluated by a one-sided paired t-test or its non-parametric alternative, if necessary. A *p* value of ≤0.05 was considered as statistically significant. Effect sizes (Cohen’s d) were calculated for differences between the two groups and for changes from baseline to post-intervention. A group-by-time interaction was examined for number of valid days and mean daily step count during the intervention period using cumulative link mixed models and linear mixed-effects models, respectively.

For the purpose of the mean daily step count, at least four valid days (at least 8 h with step count above zero) were required. If there were fewer than 4 valid days within the 7-day measurement period, additional valid days immediately after this period were added until 4 valid days were reached. All statistical analyses were performed using the statistical package R (version 3.3.3).

### Qualitative analysis

To improve the recruitment activity of the GPs, it is recommended to use qualitative research to identify and overcome barriers to recruitment and reduce the clinical workload associated with participation in clinical trials [60]. Therefore, we conducted a qualitative analysis of structured interviews performed with the 4 participating GPs after the end of the trial but before they became aware of the study’s results. The interviews were based on a topic guide focused on the feasibility of the trial and how to improve it; specifically, it comprised topics such as screening and addressing the patients, the recruitment procedure, dealing with patients’ refusal, the burden of the baseline assessment, thoughts regarding the follow up assessment, interference with their workflow, and the role of pedometers in promoting PA. The interviews were recorded and transcribed verbatim. The coding and the thematic analysis were performed by the main researcher.

## Results

### Feasibility of the recruitment procedure

The patients were recruited opportunistically, i.e., they were approached by their GP during their routine preventive visits. This procedure, though feasible, appeared to be relatively slow and inefficient. A total of 79 eligible patients from four general practices were addressed to participate in the study. Of those 79, about every second patient refused to participate (their reasons are depicted in Fig. 1), resulting in 37 recruited patients. On average, 0.63 (± 0.36) patients were recruited per week of the active recruitment. Of the 37 recruited, 23 (62%) patients were randomized. The reasons for not randomizing the recruited patients are summarized in the CONSORT flow diagram (Fig. 1).

### Patients’ adherence and engagement

Once randomized, the patients manifested high adherence to the study protocol and the PEMAIL group also exhibited a high level of engagement with the email counseling. All randomized patients completed the study and were included in the analysis.

**Fig. 2:**
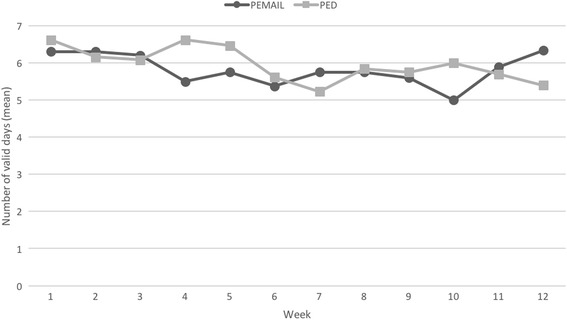
Adherence to pedometer wear during the intervention period. The effect of time was significant (*P* = 0.008), whereas the effect of group was not

Patients wore the pedometer on 83% (± 20) of the days during the 12-week intervention period. There was no significant difference between the groups in the number of valid days (i.e. days in which pedometer was worn for at least 8 h). The cumulative link mixed model revealed a significant effect of time for both groups for the entire intervention period with the highest number of valid days in the first week post-randomization (Fig. 2). However, from the third week on, there was no significant effect of time on the number of valid days in either of the group anymore. Technical issues were frequent during the study: 10 (43%) patients had their pedometer defunct for at least 1 day (11 days on average) due to technical issues (flat battery, syncing troubles).

Patients in the PEMAIL group were sent, on average, 6.7 (± 1.3) counseling emails during the intervention period. All PEMAIL patients actively participated in email communication and, on average, they responded to 46% (± 22) of the emails they received. There was no time-dependent change in the probability of responding to a counseling email during the intervention period.

### Potential efficacy of the interventions

Though the pilot randomized controlled trial was not powered to demonstrate significant differences between the groups, it has suggested that adding email counseling to a pedometer-based intervention might potentially increase the efficacy of such an intervention. Baseline characteristics of 23 randomized patients (11 females, 12 males) are summarized in Table 1. There were no significant differences between the two groups, and the baseline characteristics of the non-randomized patients were not significantly different from those who were randomized. Interestingly, the mean body mass index of the randomized patients was 33, indicating that GPs preferentially recruited overweight and obese patients (only 3 out of 23 randomized patients had a body mass index below 25). This is also reflected in the high waist and hip circumferences of the randomized patients. Of note is the equal proportion of men and women, which is atypical for lifestyle interventions.

**Table 1:** Baseline characteristics of study participants, mean (SD)

	Pedometer-plus-email (*n* = 10)	Pedometer-alone (*n* = 13)
Age (yr)	44 (10)	39 (9)
BMI (kg/m^2^)	33 (7)	33 (8)
Females (%)	30	62
Systolic blood pressure (mm Hg)	133 (9)	130 (18)
Diastolic blood pressure (mm Hg)	89 (10)	83 (15)
Waist circumference (cm)	114 (17)	102 (17)
Hip circumference (cm)	116 (10)	115 (17)
Steps per day	5034 (1431)	5050 (1393)

**Fig. 3:**
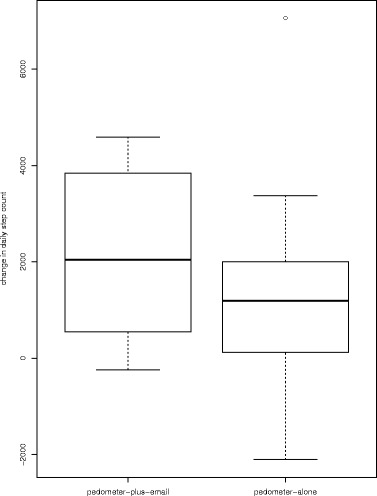
Changes in the number of steps per day from baseline to post-intervention. The difference between groups was not significant (*p* = 0.36)

**Fig. 4:**
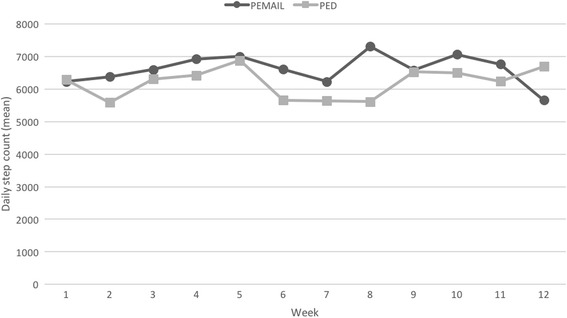
Mean daily step count during the intervention period. No effect of group or time was observed

Both groups showed a significant increase in the average number of daily steps (Fig. 3). The increase was greater in the PEMAIL group (2119 ± 1761 vs 1336 ± 2283, effect size 0.38), but the difference (783) was not significant. To detect this difference in a future trial, with a power of 80% using a 2-sided 0.05 significance level (alfa), 108 subjects in each arm would be needed. There was no group- or time-dependent change in the mean daily step count found during the intervention period (Fig. 4), which suggests that both groups increased their daily step count at the start of the intervention and then maintained it at the same level, despite the recommendation to increase their daily steps gradually. There were no differences between groups in any of the secondary outcomes.

When the two groups were analyzed as a whole, there was a significant improvement from T0 to T12 in daily step count, body mass, waist circumference, and systolic blood pressure. With the exception of change in daily step count, the effect sizes of these improvements were small or very small (Table 2).

**Table 2:** Baseline (T0) and post-intervention (T12) values of both groups combined, mean (SD)

	T0	T12	Change	*p* value	Cohen’s d
Steps per day	5043 (1377)	6719 (2359)	1676 (2066)	.0004	.87
Body mass (kg)	102.8 (21.7)	101.7 (21.6)	−0.7 (1.8)	.044	.05
Systolic blood pressure (mm Hg)	131.5 (14.3)	128.0 (12.4)	−3.5 (9.4)	.045	.26
Diastolic blood pressure (mm Hg)	85.5 (12.9)	83.7 (8.3)	−1.8 (9.7)	.193	.16
Waist circumference (cm)	107.2 (17.7)	105.4 (17.2)	−1.7 (4.0)	.029	.11
Hip circumference (cm)	115.4 (14.5)	114.8 (14.0)	−0.6 (5.0)	.292	.04

### Lessons learned from the qualitative research

Several specific topics emerged from the interviews with GPs that can influence the design of future trials regarding the recruitment process, intervention, and outcomes.

#### Recruitment

Most GPs believed that the opportunistic recruitment by a physician is more appropriate for the study than the systematic recruitment using email or post mail. Even the nurse was not regarded as an appropriate person to approach the patients. They also mentioned that remuneration for the GPs could increase their motivation to recruit patients. All GPs agreed that the preventive visits (i.e. general checkups) are a good opportunity to recruit patients because they can spend more time explaining the study, and it is natural to discuss life style changes during these preventive visits. When patients expressed a lack of interest in participating in the study, the GPs did not try to convince them, as they supposed these patients would be non-adherent further in the study. Even though they considered the recruitment procedure to be a simple one, they often deliberately avoided approaching suitable patients due to time pressure. Despite the broad eligibility criteria of the study, the GPs did a considerable amount of patient pre-selection. They typically addressed patients with obesity, diabetes, hypertension, and depression and anxiety, because they felt that these patients would be more prone to participate in the study. The GPs were well aware of the health benefits of PA in sedentary but otherwise healthy people that were eligible for the study; in spite of that, they were reluctant to recruit them because they were afraid of refusal.

#### Intervention

Technical issues related to pedometers, troubles with uploading step count data, and insufficient technical support were criticized by all GPs. They warned that these issues negatively influenced patients’ adherence to the study protocol, but also threatened their own reputations as patients tended to attribute these troubles to the GP who recruited them to the study. One GP described a negative experience with several of her patients who refused to participate in the study as they did not like the idea of being monitored and supervised. At one point, she admitted that she personally would not be happy if someone else were “watching and judging” her.

#### Outcomes

While GPs appreciated that the study protocol was relatively simple to follow, they suggested adding other secondary outcomes when designing a future trial; specifically, they mentioned serum lipid profile and blood sugar levels. On the other hand, the GPs questioned the relevance of assessing hip and waist circumferences, pointing out that such measurements are rather subjective, and that their changes are more relevant to diet than to PA.

## Discussion

Email counseling may be an effective approach for increasing the effectiveness of pedometer-based walking interventions delivered in primary care settings. Unfortunately, this approach has never been tested in a randomized controlled trial and little work has been done to provide a basis for designing such a trial. This pilot study indicates that adding email counseling to a pedometer-based intervention might yield additional benefits in terms of PA levels. The study also showed that patients recruited opportunistically during preventive visits to their GP demonstrate excellent adherence to wearing the pedometer and high levels of engagement with email counseling. This pilot study has also identified several issues that need to be addressed when designing future trials, namely the relatively slow and inefficient recruitment process, selective recruitment, technical issues, and the optimization of outcome measures.

### Results in the context of other literature

A limited body of literature suggests that a well-designed robust counseling protocol can potentiate the pedometer’s effect on PA levels, as has been shown in the PACE-UP trial where a pedometer plus three individually-tailored practice nurse consultations were more effective at increasing PA levels in 1023 physically inactive 45- to 75-year-olds at 3 months than pedometer alone distributed by post mail. However, 12 months after the start of the trial, the difference between both intervention groups disappeared, though they were both still significantly better in daily step-count and time spent doing moderate-to-vigorous PA than a control group that received usual care [21].

Compared to the PACE-UP trial, the increase in the daily step count in our study was substantially higher, which can be explained by lower baseline levels of PA in our study (5043 vs 7478 steps per day) with more room for improvement. Unlike the PACE-UP trial, our study detected small but significant improvements in body mass and waist circumference from T0 to T12, which might be related to a higher proportion of overweight and obese patients, but is also consistent with other pedometer-based interventions [14].

In general, the improvements in the daily number of steps observed in our study were higher than those reported in recent pedometer-based trials in primary care [19, 23]. For example, in a large trial with 571 primary care patients at risk of type 2 diabetes, a pedometer-based intervention supported with an initial 3-h group-based structured education program only increased the mean daily step count by 411 after 12 months compared to control group [19]. Difficulties in maintaining PA in the long term and the relatively high baseline PA levels (6585 steps per day) might have both contributed to the small effect of that intervention. Indeed, greater improvements (1029 steps per day) were demonstrated in another primary care trial with lower baseline PA levels (4771 steps per day) and a shorter follow-up period (8 weeks), despite no additional counseling component; of note is that this study used a step-counting mobile application instead of a pedometer device [23].

One of the strengths of our study is that we objectively assessed subject adherence to wearing the pedometer on a daily basis. This is a very important factor because low adherence (i.e. failure to use the pedometer daily) can hinder what would be an otherwise well-designed intervention. In spite of that, published data on adherence to pedometer wear are almost nonexistent. One study noted that 25 overweight or obese postmenopausal women wore the pedometer on 80% of intervention days during a 16-week intervention [61]. Despite minor differences in the intervention period from our protocol (12 vs 16 weeks) and methodology (valid day defined as 8 vs 10 h), this number is very close to the 83% that we observed in our study.

A unique feature of our study is that it reports on patients’ engagement with email counseling. One of the few studies that also reported on patients’ engagement with email counseling compared a complex web-based intervention for weight loss (including self-monitoring with a pedometer) alone or in combination with email counseling. In that study, no differences were observed between groups in objectively assessed PA, in spite of the high level of engagement: during the first 6 months, 89% of participants sent email responses, even though they were not required to do so [42]. As that study did not report on the total percentage of emails that were answered, our study builds on this by reporting that nearly half (46%) of all emails were responded.

One of the objectives of our pilot study was to explore the feasibility of the recruitment procedure because the success of research in primary care often depends on the recruitment of the target number of participants; indeed, many RCTs fail to recruit the actual target number [62]. Based on previous research, we have chosen opportunistic recruitment in which patients are approached while attending the practice, as this approach was associated with less time to target recruitment compared with systematic recruitment when patients are selected from practice lists and approached by post mail [63]. In our study, opportunistic recruitment was less successful, which might be attributed to the fact that, unlike in the study by Warren et al. where patients were approached by a researcher, it was the GP who personally approached the patients during routine preventive visits. Participant eligibility based on self-reported physical inactivity could also contribute to a lower than expected number of patients, as people tend to overestimate their level of physical activity [64, 65], thus effectively excluding themselves from the study.

On the other hand, once randomized, all patients in our study completed the 12-week follow-up which is in contradiction with the high dropout rate after 12 weeks (28.8%) that was observed by Warren et al. This may be explained by our pre-randomization procedure that demanded patients to upload their pedometer data to a website, which 27% failed to do. Therefore, it may be that only highly motivated patients were randomized and ultimately participated.

An additional reason for choosing opportunistic recruitment was our assumption that it would reduce the self-selection bias typical for systematic recruitment, where only those patients ready for a behavioral change respond, thus decreasing the external validity of a study. While our assumption was more or less confirmed, as only about half of the approached patients refused to participate in the study (for comparison, in the PACE-UP trial, 85% of systematically invited patients either did not respond or refused to participate [21]), the opportunistic recruitment strategy introduced a different type of a selection bias caused by GPs who only approached a small proportion of their patients who were eligible for the study. This selection bias is supported by the unexpectedly high body mass index of our randomized patients and is also confirmed in our qualitative analysis of the GP interviews. Our finding is in line with a Cochrane review that concluded that clinicians are concerned that their relationship with the patients would be adversely affected by participating in a trial [66].

In spite of this ambiguous experience with opportunistic recruitment, the qualitative analysis revealed that GPs still consider opportunistic recruitment during the routine preventive visits as an appropriate way to recruit participants, a view that is also supported in the literature [67]. However, to speed up the recruitment process, a mix of opportunistic and systematic recruitment should be considered when designing the main trail.

### Study strengths and limitations

The strengths of this pilot study are (a) the involvement of 4 general practices representing various urban areas, (b) a balanced representation of men and women, (c) the detailed reporting of patients’ adherence and engagement, (d) the complementation of quantitative outcomes with a qualitative analysis.

The limitations of the study include the selection bias towards overweight and obese patients and the high number of recruited participants that were not randomized. The reasons for non-randomization mainly include three factors, each representing approximately one-third of such patients: (1) technical issues that hindered the upload of data, (2) patients were excluded due to achieving > 8000 steps a day at T0, (3) patients stopped communicating after they were recruited. The technical issues should be resolved in a future trial by using another type of pedometer. The exclusion of patients achieving > 8000 steps a day at baseline is pre-specified in the eligibility criteria, and their number corresponds to the expected positive predictive value of the screening question [54], so it is a limitation that cannot be addressed. The non-communication of the patients might be related to the opportunistic recruitment strategy, where patients who would not normally participate when approached by post mail are too shy to refuse participation when confronted face-to-face with their GP, despite not feeling committed to cooperate once they leave the practice. A more neutral way of extending the invitation to participate and avoiding any inadvertent push or forceful recruitment strategies might resolve this issue.

The selection bias towards overweight and obese patients is a more serious weakness that limits the external validity of this study. Although GPs understood that inactive but otherwise healthy patients could benefit from increasing PA levels, they preferentially recruited obese patients as they believed that these patients would be less likely to reject the invitation. The fear of rejection has also been described elsewhere [66], and thus it is not likely that better training would change the GPs recruitment behavior. Therefore, another effective strategy, e.g., stratified sampling, should be adopted to eliminate this bias in a future trial.

Another limitation of the study is the small sample size, which has implications not only for the insufficient power of the trial, but also for the eventual scaling up of the intervention. Specifically, the counseling emails in this pilot study were all individually tailored by the main researcher and thus the intervention cannot be simply translated into real world practice. This issue need to be addressed in future trials, for example by training physiotherapists or nutritional therapists to provide the email counseling, or by employing automated computer-tailored counseling.

### Implications for practice

This is the first study to evaluate the additional benefit of email counseling on top of a pedometer-based intervention aimed at increasing PA. Our data generally agree with previous studies of face-to-face or phone counseling added to a pedometer and extends their findings to email counseling. The study was intended as a pilot study and yielded important findings supporting the feasibility of future trials, specifically:

1. Patients manifest high adherence to wearing the pedometer daily for the period of at least 12 weeks.
2. The study protocol is easy to follow both for GPs and patients, as indicated by 0% attrition during a 12-week period.
3. Email counseling is well accepted by patients who manifested high engagement, as demonstrated by their responses to the counselor’s emails.
4. Though not sufficiently powered to demonstrate superiority of the PEMAIL group over the PED group, the study indicated that email counseling might have the potential to increase the efficacy of a pedometer-based intervention; the efficacy data have been used to calculate sample size of a future trial.

On the other hand, the study has also revealed possible areas for improvement:

1. The inefficiency of the opportunistic recruitment procedure and the selection bias introduced by GPs, who preferentially approached overweight and obese patients, need to be addressed to ensure that future trials have implications for public health, possibly by finding the right mix of opportunistic and systematic recruitment and implementing a stratified sampling method. Financial incentives for participating GPs to recruit more patients should be considered as well, carefully weighing their pros and cons [68].
2. The pedometer used in this study should be replaced by a more user-friendly, bullet-proof technology to avoid technological failures and subsequent annoyance for patients and GPs.
3. Additional outcomes could be possibly introduced (serum lipid profile, blood sugar levels) while keeping the study protocol simple and easy to follow.
4. A longer follow-up of at least 12 months is generally required in PA interventions to assess the maintenance of the intervention effect [6].

## Conclusions

To the best of our knowledge, this is the first study demonstrating that adding email counseling to a pedometer-based intervention in a primary care setting is feasible and might have the potential to increase the efficacy of such an intervention. Thus, the study provides important information for conducting future randomized controlled trials assessing the additional benefit of email counseling added to a pedometer-based intervention delivered in general practice. If shown to be effective, dissemination of such an intervention in primary care will help GPs better fulfill their role as promoters of healthy behavior: a role that is perceived as fundamental by both GPs and their patients.

## Abbreviations

GP: General practitioner
PA: Physical activity
PED: Pedometer-alone group
PEMAIL: Pedometer-plus-email group
